# One-Pot Enzymatic Production of Lignin-Composites

**DOI:** 10.3389/fchem.2018.00124

**Published:** 2018-04-20

**Authors:** Sabina Ion, Cristina Opris, Bogdan Cojocaru, Madalina Tudorache, Irina Zgura, Aurelian C. Galca, Adina M. Bodescu, Madalin Enache, Gabriel-Mihai Maria, Vasile I. Parvulescu

**Affiliations:** ^1^Department of Organic Chemistry, Biochemistry and Catalysis, Faculty of Chemistry, University of Bucharest, Bucharest, Romania; ^2^Laboratory of Optical Processes in Nanostructured Materials, National Institute of Materials Physics, Magurele, Romania; ^3^Laboratory of Multifunctional Materials and Structures, National Institute of Materials Physics, Magurele, Romania; ^4^Faculty of Food Engineering, Tourism and Environmental Protection, Research Center in Technical and Natural Sciences, “Aurel Vlaicu” University, Arad, Romania; ^5^Institute of Biology Bucharest of the Romanian Academy, Bucharest, Romania

**Keywords:** bio-composites, oxi-copolymerization, monolignols, peroxidase enzyme, lignin

## Abstract

A novel and efficient one-pot system for green production of artificial lignin bio-composites has been developed. Monolignols such as sinapyl (SA) and coniferyl (CA) alcohols were linked together with caffeic acid (CafAc) affording a polymeric network similar with natural lignin. The interaction of the dissolved SA/CA with CafAc already bound on a solid support (S_C2_/S_C6_-CafAc) allowed the attachment of the polymeric product direct on the support surface (S_C2_/S_C6_-CafAc-L_1_ and S_C2_/S_C6_-CafAc-L_2_, from CA and SA, respectively). Accordingly, this procedure offers the advantage of a simultaneous polymer production and deposition. Chemically, oxi-copolymerization of phenolic derivatives (SA/CA and CAfAc) was performed with H_2_O_2_ as oxidation reagent using peroxidase enzyme (2-1B mutant of versatile peroxidase from *Pleurotus eryngii*) as catalyst. The system performance reached a maximum of conversion for SA and CA of 71.1 and 49.8%, respectively. The conversion is affected by the system polarity as resulted from the addition of a co-solvent (e.g., MeOH, EtOH, or THF). The chemical structure, morphology, and properties of the bio-composites surface were investigated using different techniques, e.g., FTIR, TPD-NH_3_, TGA, contact angle, and SEM. Thus, it was demonstrated that the SA monolignol favored bio-composites with a dense polymeric surface, high acidity, and low hydrophobicity, while CA allowed the production of thinner polymeric layers with high hydrophobicity.

## Introduction

Together with cellulose and hemicellulose, lignin is one of the most abundant natural polymers (Zakzeski et al., 2010). As an effect of the excessive valorization of the natural resources, the pulp-paper and bio-refining industries are important providers of lignins (Lora and Glasser, 2002). However, the papermaking industry produces annually over 50 million tons of lignin wastes that are mainly used as an energy source (by direct combustion). Today, only 2 wt% of it is used for the polymeric industry (e.g., production of phenolic resins, polyurethane foams, bio-dispersants, or epoxy resins) (Gosselink et al., 2004b). Additionally, the wastes of the paper-pulp industry generate serious environmental pollution concerns. So that, an improvement of the market competitiveness of bio-refining industry is necessary. Accordingly, new perspectives for the valorization of the lignin are required.

The general perception on the lignin is that of a “renewable chemical resource” formed from the assembling of functionalized aromatic entities with phenolic hydroxyl, alcoholic hydroxyl, carboxyl, or methoxy groups (Xiong et al., 2015; Aro and Fatehi, 2017). It presents an amorphous polymeric structure assumed to derive from up to three monolignols, e.g., coniferyl alcohol (CA), synapyl alcohol (SA), and coumaryl alcohol. These monolignols are incorporated in phenylpropanoid units expressed in varied modes such as guaiacyl, syringyl, and *p*-hydroxyphenyl connected by ether and carbon-carbon bonds in a complex three dimensional polymeric network (Nair et al., 2014). Thus, what is named lignin corresponds to a material with a large variety of chemical structures in which the above mentioned entities exist in various proportions. The differences are the direct consequence of its origin (type of the plant species, climate, geographical location) and the extraction process (Gosselink et al., 2004a).

As a bio-polymer, there are many potential value-added applications of lignin with significant impact on industry (Lee and Wendisch, 2017). Derivatization of lignin is often chosen as alternative leading to functionalized bio-polymers with role of the dispersant for cement, pesticide, coal-water slurry, rubber-based material, component of animal feed, surfactants, additive in oil drilling, stabilizers in colloidal suspensions, etc (Xiong et al., 2015; Aro and Fatehi, 2017). The lignin derivatives may also acquire antioxidant, antiviral, antibiotic, and/or anticarcinogenic activities (Yamamoto et al., 1997; Vinardell et al., 2008; Nair et al., 2014). Lignin has been used as alternative to phenol in phenolic resins and also in the composition of thermoplastic polyesters, polyurethanes, active carbons, and carbon fibers (Stewart, 2008; Thanh Binh et al., 2009; Nair et al., 2014). Additionally, lignin-based composites appeared as another low cost eco-friendly reinforcement attractive alternative (Morandim-Giannetti et al., 2012; Pupure et al., 2013; Qian et al., 2014; Thakur et al., 2014). They confirmed as important ingredients for composites with various properties like thermoplastic polymers (Pucciariello et al., 2004; Barzegari et al., 2013), thermosetting polymers (Yin et al., 2012; Stanzione et al., 2013), rubbers (Setua et al., 2000; Kramárová et al., 2007), or foam-based materials (Del Saz-Orozco et al., 2012; Luo et al., 2013). For this purpose, lignin and its derivatives were incorporated in bio-composites using both chemical or/and physical methods leading to homogeneous lignin particles (Nair et al., 2014) or colloidal spheres produced through self-assembly of acetylated lignin (Qian et al., 2014). However, such a valorization of lignin is still restricted due to two main reasons: its relative low reactivity and high heterogeneity of the polymeric mixture (Qu et al., 2015). Therefore, in the last years several chemical methods have been reported to enhance lignin reactivity, e.g., methylation, demethylation, acetylation, etc (Hu et al., 2011). Additionally, the lignin modification has been achieved *via* catalytic and solvent-free methods (e.g., graft copolymerization of lactides to lignin catalyzed by triazabicyclodecenes) (Chung et al., 2013). However, the heterogeneity of the polymeric mixture of lignin is still a challenge today.

In this study, we investigated the production of lignin-composites (bio-composites) using monolignol fractions (e.g., SA or CA) with the aim to find another route for the valorization of lignin. There are evidences that lignin can be efficiently disrupted into a cocktail of monomers and oligomers (Lee et al., 2013; Opris et al., 2016, 2017). The produced fragments can be re-combined leading to an artificial lignin structure with enhanced homogeneity compared to the original lignin (Opris et al., 2018). In this study, particles functionalized with CafAc were used as solid supports allowing the synthesis of bio-composites. Therefore, monolignols can be directly oxi-polymerized on the particles surface involving the CafAc as co-monomer. Accordingly, the coverage process was called oxi-copolymerization. The use of monolignols instead of whole lignin molecule ensures a better control of the composition of the polymeric layer providing to a good structural homogeneity of the polymeric material.

Usually, the functionalization of the particles surface (e.g. silica particles) by either physical or chemical methods requires a prior modification before covering with the polymeric layer. Most of the modifiers are derived from the fossil resources increasing the cost of these materials but also the toxicity (Zou et al., 2008). In this study, the particles surface (methacrylate) was functionalized with a natural modifier, i.e., CafAc (CafAc occurs frequently in fruits, grains, *Salvia* species) (Hao et al., 2015). Also, the polymeric layer (artificial lignin) covering the surface is bio-derived, mimicking the original lignin. In accordance to its characteristic structure and properties, the lignin-based polymer may afford the required modification of the particles for further bio-applications (e.g., carrier/support for enzymes). Additionally, the lignin bio-composites can provide an important example of a high value utilization of lignin residues.

The oxi-copolymerization of monolignols reported in this study for the synthesis of bio-composites was designed as a biocatalytic process where a peroxidase enzyme assisted the oxidation of both monolignols (SA or CA) and CafAc by means of H_2_O_2_. The developed system acts based on an one-pot approach combining the oxi-copolymerization of SA/CA with CafAc, and the attachment of the resulted polymer on the support surface. From our best knowledge, it is the first time when a lignin-composite (bio-composite) with controlled and reproducible composition is prepared based on one-pot approach. Additionally, the influence of a co-solvent (e.g., MeOH, EtOH, THF) on the oxi-copolymerization process has also been investigated. The production of the bio-composites was monitored using spectrophotometric as well as Folin-Ciocalteu analysis. Also, detailed characterization of the lignin-composites was performed using different techniques, e.g., FTIR, TPD-NH_3_, TGA, contact angle, and SEM.

## Experimental

### Chemicals and solutions

The oxi-copolymerization process was performed with 2-1B mutant of versatile peroxidase original from *Pleurotus eryngii*, expressed in *Saccharomyces cerevisae* (12.95 U mL^−1^ enzyme activity) (Garcia-Ruiz et al., 2012; Molina-Espeja et al., 2014, 2015). 2-1B mutant was provided by Dr. Miguel Alcalde (Institute of Catalysis, CSIC, Madrid, Spain). The solid support of bio-composites (S_C2_–amino C2 methacrylate, ECR8309F and S_C6_–amino C6 methacrylate, ECR8409F) was kindly offered by the Purolite Life Sciences Company. Both supports were constituted from the beads (150–300 μm of diameter) originally functionalized with -NH_2_ groups using methacrylate cross-linkers.

Ten milli molars of PBS (phosphate buffer saline) solution (pH = 7.4) was used as aqueous buffer solution. Its composition consisted of: 8 g NaCl, 0.2 g KCl, 1.43 g Na2HPO_4_ × 2H_2_O and 0.34 g KH_2_PO_4_ in 1 L distilled water. 10 mM MES (2-(N-morpholino)ethanesulfonic acid) buffer (pH = 4.7) was prepared by dissolving a corresponding MES mass in distilled water followed of NaOH addition for adjusting the pH value of the solution.

CA, SA, CafAc, 1-ethyl-3-(3-dimethylaminopropyl)carbodiimide (ECD), 30 wt% solution of hydrogen peroxide (H_2_O_2_), methanol (MeOH), ethanol (EtOH), and tetrahydrofuran (THF) were of analytic purity and purchased from Sigma-Aldrich. Stock solutions of monolignols (e.g., CA and SA) with concentration of 10 mg mL^−1^ were prepared in MeOH.

### S_C2_/S_C6_ functionalization

For the attachment of the lignin polymer, the solid supports S_C2_ and S_C6_ were functionalized with CafAc in order to ensure the phenolic structures on the particles surface. 0.2 g of S_C2_/S_C6_ support were dispersed in 10 mL solution containing 10 mg mL^−1^ CafAc in MES (10 mM, pH = 4.7). 0.96 g of EDC was added in the suspension under continuous shaking for activating the -COOH groups of CafAc in order to interact with—NH_2_ groups on the support. Coupling of CafAc on support surface was performed over the night, at room temperature under gentle agitation. Then, the functionalized particles (S_C2_-CafAc and S_C6_-CafAc) were separated by centrifugation and washed several times with MES buffer and distilled water.

### Bio-composites preparation

The bio-composites construction was performed in one-pot system by enzyme oxi-copolymerization of monolignols (e.g., CA or SA) directly on the surface of the previously prepared supports (S_C2_/S_C6_ functionalized with CafAc, section S_C2_/S_C6_ functionalization). One hundred microliters of monolignol stock solution (10 mg mL^−1^ SA/CA in MeOH) were diluted with 300 μL PBS (10 mM, pH 7.4). 10 mg of functionalized support (S_C2_-CafAc or S_C6_-CafAc) were added in the solution followed by the addition of 10 μL H_2_O_2_ (30%) and 50 μL 2-1B peroxidase mutant (H_2_O_2_:enzyme molar ratio of 1760:1). The reaction mixture was incubated at 40°C in a thermo-shaker (100 rpm) over night. For comparison, the monolignols polymerization was also performed in the absence of the functionalized support (homogeneous system).

**Scheme 1:**
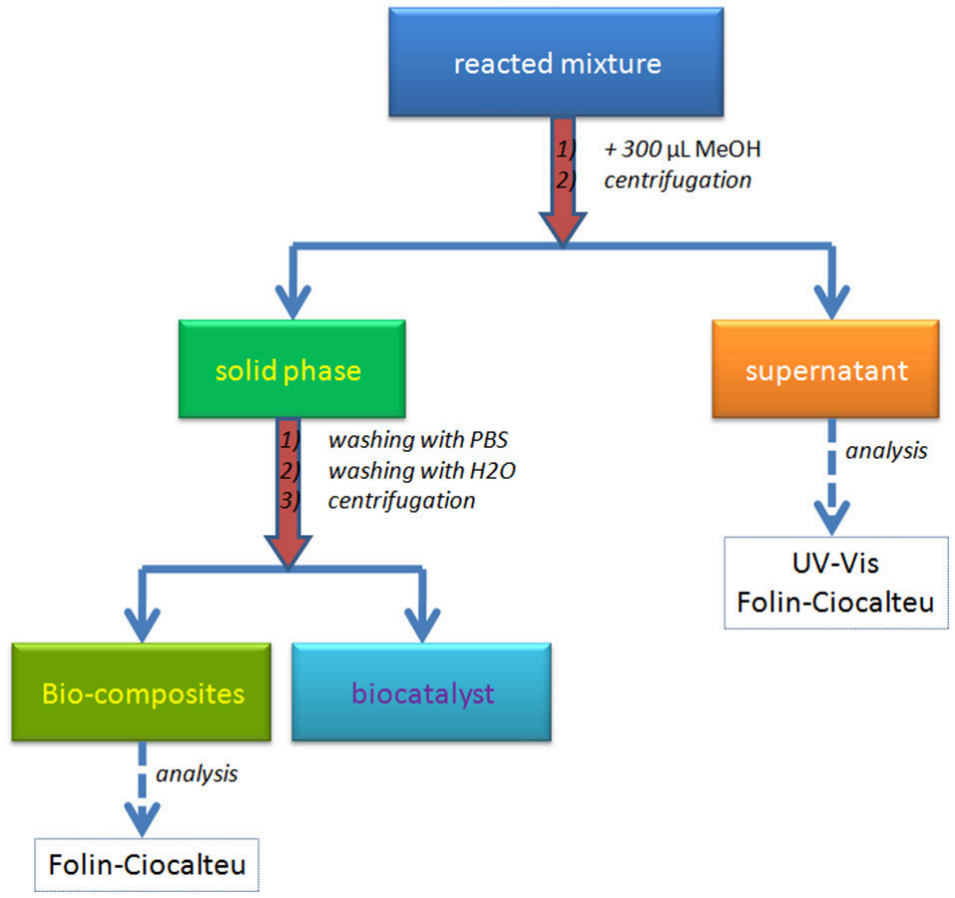
Protocol for separation of bio-composites from the reacted mixture.

The separation of the bio-composite was performed using a set up protocol detailed in Scheme 1. The reacted mixture was treated with 300 μL MeOH for biocatalyst precipitation and solubilization of unattached oligomers. The centrifugation of the new mixture allowed the separation of the two phases: the liquid phase (supernatant) with unreacted monolignols and/or oligolignols, and the solid phase containing the bio-composites and precipitated biocatalyst. The supernatant was analyzed using UV-Vis and Folin-Ciocalteu methods. While the UV-Vis analysis allowed the determination of the aromatic content, the Folin-Ciocalteu method permeated the detection of the phenolic -OH groups. The solid phase was washed consecutively with PBS and distilled water in order to remove the precipitated enzyme (biocatalyst) from the bio-composites surface. The centrifugation step allowed the separation of the bio-composites and the recovery of the enzyme as a PBS solution. Bio-composite surface was investigated based on Folin-Ciocalteu approach.

### Analysis of reacted mixture

UV-Vis method was used for the determination of mono-/oligo- lignols (phenolic derivatives) in the liquid phase (Scheme 1). The sample absorbance was read at 280 nm with a Specord 250 (Analytik Jena). The conversion of monolignols based on the oxi-copolymerization process was calculated using the absorbance of the solutions before and after the reaction. Folin-Ciocalteu analysis was performed for the determination of free -OH groups on the aromatic ring (Singleton et al., 1999). The protocol was adapted for the investigation of the bio-composites and the liquid phase (Scheme 1). Twenty microliters of sample solution (supernatant or 10 mg/mL bio-composites in MeOH) were dispersed in 1.5 mL of distilled water and the resulted mixture was enreached with 100 μL Folin-Ciocalteu reagent and 300 μL saturated solution of sodium carbonate as supporting medium (adjusting the pH of solution to a basic value). After 2 h incubation time, the mixture was centrifuged and the supernatant absorbance was read at 765 nm.

GPC analysis was performed for the determination of the average molecular weight of the polymers produced in the absence of the functionalized support. The analysis approach was detailed in our previous report (Opris et al., 2018). Thus, the GPC analysis was carried out using an Agilent Technologies instrument (Model 1260) equipped with two columns (Zorbax PSM 60-S, 6.5 × 250 mm, 5 μm, and Polargel-M, 300 × 7.5 mm) and a multidetection unit (Refractive Index, Light Scattering, and Viscosity detectors). Experimental conditions were set up at 1 mL min^−1^ THF as mobile phase, 100 μL injection volume of sample, and temperature of the detectors and columns of 35°C. The calibration of the GPC system was performed using polystyrene standards in the range of 162–10,000 g mol^−1^. The Agilent GPC/SEC Software (Version 1.1, Agilent Technologies) was utilized for the determination of the average molecular weight (MW).

### The characterization of the bio-composites

FTIR spectra of bio-composites and simple/functionalized supports were recorded using Vertex 70 (Bruker, Ettlingen, Germany) spectrophotometer equipped with the Total Attenuated Reflectance cell in the range of 600–2000 cm^−1^. Sixteen scans were collected with a resolution of 4 cm^−1^ in the range of 600–4000 cm^−1^.

The total acidity was evaluated by temperature-programmed desorption of ammonia (TPD-NH_3_) using a Micromeritics Chemisorb 2750 instrument. Before NH_3_ desorption, the samples were heated to 80°C (20°C min^−1^) in 30 mL high pure Helium flow. Subsequently, the samples were cooled down to room temperature in helium flow. NH_3_ adsorption was performed under ambient conditions and saturation for about 60 min in a flow of 10% ammonia in Helium (30 mL min^−1^). Then, the samples were purged in a Helium flow until a constant baseline level was attained. The desorption of NH_3_ was carried out with the linear heating rate (10°C min^−1^) in a flow of Helium until 300°C.

The termo-gravimetric analysis (TGA) was performed with a Shimadzu instrument (SDT Q600) in order to determine the thermo stability of the bio-composite according to the original/functionalized support. Maximum 10 mg of sample were used. The analysis was carried out at increasing temperature with a rate of 10°C min^−1^ in the range of 30–600°C under N_2_ atmosphere.

Static contact angle of bio-composites was measured with a Drop Shape Analysis System, model DSA100 (Kruss GmbH). The sample was placed on a horizontal stage, under the tip of a water-dispensing disposable blunt-end stainless steel needle with an outer diameter of 0.5 mm. The water droplet (1 μL) was delivered on the sample surface by the needle attached to a syringe pump controlled with a PC (through DSA3® software supplied with the instrument). The viewing camera for taking the picture was positioned to observe the droplet under an angle of about 2–3° with respect to the plane of the sample surface supporting the droplet. The tests were carried out at room temperature. The contact angle was measured by fitting a polynomial equation of second degree or a circle equation to the shape of the sessile drop. Then, the slope of the tangent to the drop at the liquid-solid vapor interface line was calculated. (Zgura et al., 2010, 2013; Popescu et al., 2011; Duta et al., 2012; Preda et al., 2013).

For scanning electron microscopy (SEM) analysis, freeze, and dried particles (e.g., bio-composites and original/functionalized supports) were examined using a Jeol instrument (JSM-6610LV). The pretreatment of the samples followed the dispersion of the particles in EtOH solution (30%) and deposition of 10 μL suspension on the microscopic blade covered with gold layer. After EtOH evaporation at room temperature, prepared blades were dried in vacuum followed by metal coating using a sputter coater (Jeol auto fine coater, JFC-1300). SEM investigations were performed under high vacuum conditions.

## Results and discussion

### Co-polymerization of monolignols for the construction of bio-composites

The concept of the one-pot synthesis of bio-composites has been described above. The oxi-copolymerization of monolignols (e.g., CA or SA) was directly performed on the supports (S_C2_ and S_C6_) surface functionalized with CafAc (S_C2_-CafAc and S_C6_-CafAc) in the presence of the 2-1B mutant of versatile peroxidase as catalyst using an adapted procedure (Opris et al., 2018). In this study CafAc used as a co-monomer afforded the oxi-copolymerization of monolignols. CA allowed the production of S_C2_/S_C6_-CafAc-L_1_ bio-composite, while the oxi-copolymerization of SA led to S_C2_/S_C6_-CafAc-L_2_.

Under the investigated homogeneous conditions, the oxi-copolymerization of monolignols (SA/CA) and CafAc led to the results presented in Table 1. A higher conversion was achieved for SA compared to CA (65.3 vs. 21.1%) leading to polymers with different molecular weights (3500 and 856 Da for SA and CA, respectively). The affinity of the biocatalyst for the monolignols can be a reasonable explanation of the system behavior. These results are in accordance with the previous report (Opris et al., 2018).

**Table 1:** Efficiency of the oxi-copolymerization process.

**Solid support**	**SA**		**CA**	
	**C (%)**	**MW (Da)**	**C (%)**	**MW (Da)**
–	65.3	3500	21.1	856
S_C2_-CafAc	71.1	–	49.8	–
S_C6_-CafAc	65.7	–	36.3	–

*Experimental conditions: 2 mg/mL monolignol (SA/CA), 1.295 U mL^−1^ 2-1B peroxidase mutant, 0.6% H_2_O_2_, and 20 mg/mL functionalized support for heterogeneous co-polymerization in PBS (10 mM, pH = 7.4); 40°C, 24 h and 100 rpm. C, conversion of the oxi-copolymerization process. MW, average molecular weight of copolymer*.

Under heterogeneous conditions, i.e., with SA/CA dissolved in liquid phase, and CafAc attached on the particles (S_C2_/S_C6_-CafAc), the monolignols and immobilized CafAc were linked together in a polymeric structure miming the natural lignin directly attached on the particles surface (Table 1). This heterogeneous design allowed to improve the conversion of monolignols compared to homogeneous system keeping the same advantage of SA vs. CA. Different conversions were also determined as a function of the functionalized support (S_C2_-CafAc and S_C6_-CafAc) demonstrating that the solid support influenced the co-polymerization process (Table 1).

Important parameters of this process are the loading of the polymeric products on the solid surface and the percent of the oligomers in the residual phase (supernatant, see Scheme 1). To determine these, both the supernatants and bio-composites were analyzed using spectrophotometric (UV-Vis) and Folin-Ciocalteu approaches (F-C) (Scheme 1). Moreover, the results were converted in the concentration of recovered monolignols (i.e., the ratio between the concentration of monolignol in/on residues/bio-composite and initial concentration of monolignol) (Table 2). The relative low difference between the F-C and UV-Vis results can be an indicative for the insignificant content of the oligolignols in the supernatant. Moreover, the UV-Vis results should be interpreted with caution because of the production of quinone derivatives as secondary products of co-oxipolymerization process. For bio-composites, the same measurements evidenced the presence of small concentration of free OH groups (around 10 times lower than in the supernatant). The participation of the OH groups into the formation of the etheric bonds of the synthetic lignin is a plausible explanation.

**Table 2:** Evaluation of supernatant content in monolignols and oligolignols using spectrophotometric (UV-Vis) and Folin-Ciocalteu (F-C) methods (recovered concentration, %).

**Solid support**		**SA**		**CA**	
		**UV-Vis**	**F-C**	**UV-Vis**	**F-C**
S_C2_-CafAc	Supernatant	28.90	24.70	58.37	52.64
	Bio-composite	–	2.78	–	5.51
S_C6_-CafAc	Supernatant	34.30	24.41	74.07	65.41
	Bio-composite	–	2.89	–	3.70

**Figure 1:**
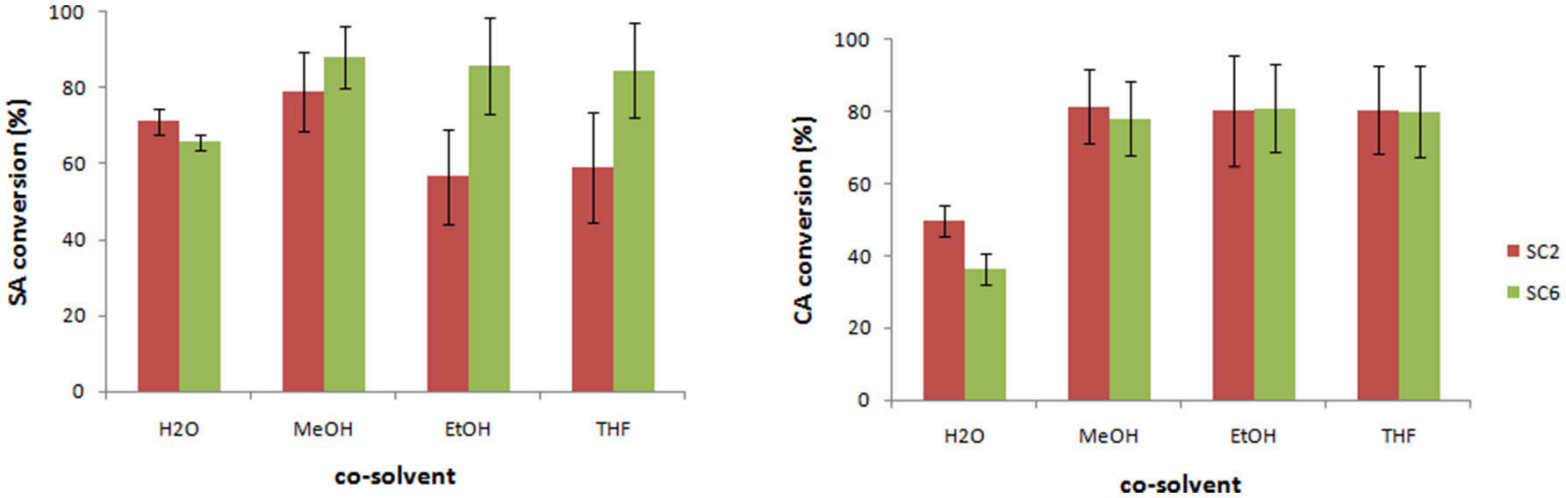
Preparation of the bio-composite in the presence of organic solvent. Experimental conditions: 2 mg/mL monolignol (SA/CA), 1.295 U mL^−1^ 2-1B peroxidase mutant, 0.6% H_2_O_2_ 20 mg/mL functionalized support for heterogeneous co-polymerization, and 6% added solvent (H_2_O, MeOH, EtOH, THF) in PBS (10 mM, pH = 7.4); 40°C, 24 h and 100 rpm. (Triplicates analysis were performed).

The effect of the co-solvent (e.g., MeOH, EtOH, THF) has also been evaluated showing a different influence depending on the monolignol type (Figure 1). For SA, the use of MeOH as co-solvent led to better results affording a higher conversion for S_C6_-CafAc support compared to S_C2_-CafAc. For CA, the presence of the co-solvent doubled the conversion of monolignols. However, the reproducibility was low, and high standard deviation has been determined for co-solvent (e.g., MeOH, EtOH, THF). In conclusion, poor reproducibility for the bio-composite production using a co-solvent enforces the use of H_2_O despite of smaller conversions.

### Characterization of the bio-composite

FTIR analysis confirmed the attachment of the artificial lignin on the support surface during the oxi-copolymerization (Figures S1–S3). Table 3 presents the differences between the spectra collected for S_C2_-CafAc-L_1_/L_2_ and S_C6_-CafAc-L_1_/L_2_, and those of the original/functionalized supports (S_C2_, S_C6_, S_C2_-CafAc and S_C6_-CafAc). The attachment of the CafAc on the solid support led to a consistent modification of the spectrum (Figure S1). The intensity of the OH band stretching at 3370 cm^−1^ on the particles surface (S_C2_/S_C6_) (Poletto and Zattera, 2013) decreased for S_C2_/S_C6_-CafAc. Additionally, new bands occurred at 1041 cm^−1^ and 1563–1564 cm^−1^ due to the presence of C-O bonds on the aromatic ring and the formation of amide bonds (CO-NH) by the attachment of CafAc on the support surface. Both functionalized supports (S_C2_-CafAc and S_C6_-CafAc) have similar FTIR spectra (Figure S1).

**Table 3:** Summary of the specific bands observed for bio-composites, original, and functionalized supports.

**Assignment**	**Band position (cm^−1^)**						
	**S_C2/C6_**	**S_C2_-CafAc**	**S_C6_-CafAc**	**S_C2_-CafAc-L1**	**S_C6_-CafAc- L_1_**	**S_C2_-CafAc-L_2_**	**S_C6_-CafAc-L_2_**
O-H stretching	3370	3361	3368	3385	3378	3392	3364
Aromatic methyl and methylene groups	–	–	–	2953	2942	2952	2943
	–	2724	2721	–	–	–	–
-CO-NH-	–	1563	1564	–	–	–	–
C-C in aromatic skeleton	–	–	–	1544	1538	1546	1540
Syringyl units	–	–	–	–	–	1120	1120
C-O deformation of aromatic ethers	–	–	–	1080	1080	1076	1076
C-O on the aromatic skeleton	–	1041	1041	–	–	–	–
C-H out of plane for guaiacyl units	–	–	–	859	859	858	859

S_C2_-CafAc-L_1_/L_2_ and S_C6_-CafAc-L_1_/L_2_ presented modified spectra in the regions of 3700–3000 cm^−1^ and 1200–700 cm^−1^ compared with S_C2_-CafAc and S_C6_-CafAc, respectively (Figure S2). The differences are summarized in Table 3. The shoulder at around 3600 cm^−1^ is attributed to an aliphatic OH group of the monolignol structure (Poletto and Zattera, 2013), while the new bands at 2952/2953 and 2942/2943 cm^−1^ were to the C-H stretching vibration of the methyl and methylene groups introduced by the SA and CA monolignols (Xiong et al., 2015). The presence of the aromatic ring has been confirmed by the bands in the range 1538–1546 cm^−1^. The new band at 1120 cm^−1^ observed for S_C2_/S_C6_-CafAc-L_2_ represents an evidence of the presence of the syringyl units detected in the polymeric layer of the bio-composites. Guaiacyl units were also detected at low intensity at 858 and 859 cm^−1^ (Poletto and Zattera, 2013). However, the band at 1264 cm^−1^ typical for guaiacyl (Poletto and Zattera, 2013; Fitigău et al., 2015) is not visible for S_C2_/S_C6_-CafAc-L_1_ due to a superposition with an already existing band at 1257 cm^−1^. Poor covering of the support with polymers from CA (Table 1) can represent another reason for the absence of this band. All bio-composites showed also absorbance bands at 1076 and 1080 cm^−1^ attributed to etheric groups (C-O-C) connecting the aromatic rings (Qu et al., 2015; Xiong et al., 2015). This is also an evidence for the production of the artificial lignin onto the bio-composite surface.

FTIR spectra of bio-composites prepared in the presence of co-solvent showed differences between MeOH and EtOH, on one side, and THF, on the other side (Figure S3). Thus, the presence of MeOH or EtOH led to structures for which the bands at 1076 and 1078 cm^−1^ were not present anymore while those at 973/949 and 858 cm^−1^ presented a dramatic decrease in the intensity. These results confirm that MeOH and EtOH inhibit the formation of the artificial lignin structures, and especially of the guaiacyl units. So, the increased conversion for the oxi-copolymerization in the presence of an organic solvent does not correspond to the formation of lignolic structures (i.e., guaiacyl units).

The acidity of the bio-composites has been evaluated from TPD-NH_3_ measurements (Table 4). The bio-composites incorporating CA have similar acidic properties with the functionalized support confirming the conservation of the phenolic OH group (NH_3_ desorption peaks at 197°C). However, the number of acidic centers has been doubled for the bio-composite showing an enrichment of phenolic OH owing of the synthetic lignin structure. CA and SA led to bio-composites with two different acidity centers (NH_3_ desorption peaks at 197 and 210°C) that were assigned to phenolic and aliphatic OH groups. Also, the total number of the acid centers was higher for SA polymer than for the corresponding CA-based bio-composite. These results fit the values of the conversion calculated for the oxi-copolymerization of the monolignols (Table 1).

**Table 4:** Acidity of bio-composites compared to the CafAc-functionalized support based on TPD-NH_3_.

**Bio-composite**	**Acidity (μmol/g)**	
	**Phenolic OH**	**Aliphatic OH**
S_C6_-CafAc	26	–
S_C6_-CafAc-L_1_	52	–
S_C6_-CafAc-L_2_	37	44

**Figure 2:**
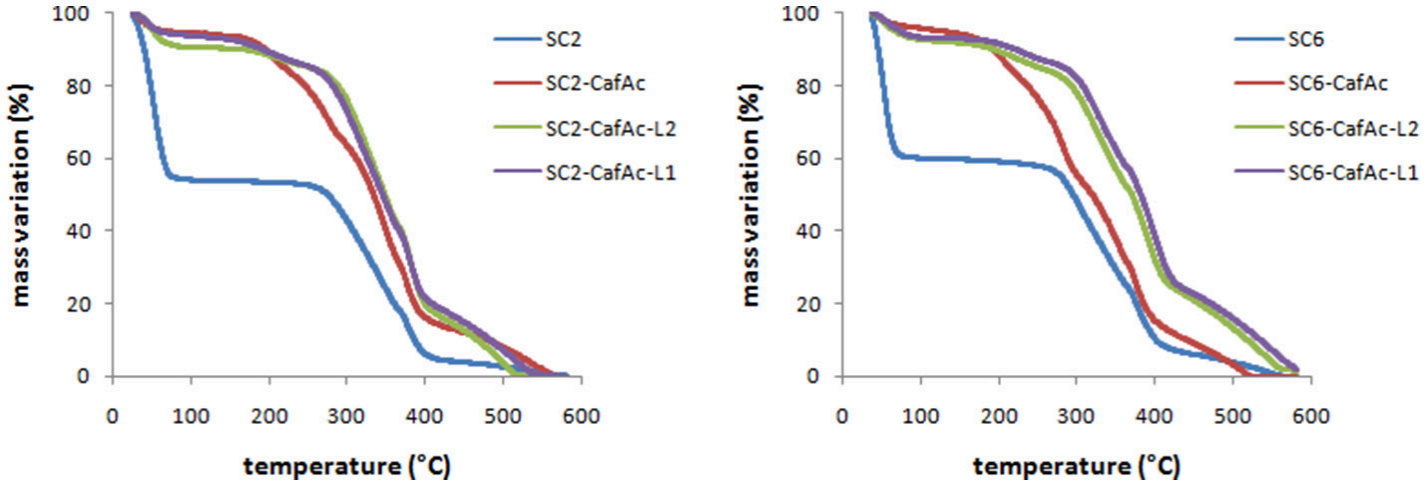
TGA diagrams of the bio-composites (S_C2_-CafAc-L_1_/L_2_ and S_C6_-CafAc-L_1_/L_2_) related to original/functionalized support (S_C2_, S_C6_, S_C2_-CafAc, S_C6_-CafAc).

The thermo-stability of produced lignin bio-composites (S_C2_-CafAc-L_1_/L_2_ and S_C6_-CafAc-L_1_/L_2_) compared to the original/functionalized supports (S_C2_, S_C6_, S_C2_-CafAc, S_C6_-CafAc) has been evaluated in the temperature range of 30–600°C (Figure 2). TGA profiles of bio-composites were quit similar. The mass loss up to 230°C is mainly due to the removal of water (<8 wt%). The differences in the shape of the profiles for the functionalized support and corresponding bio-composite may account the degradation of propanoid chain of the polymer (Strzemiecka et al., 2016). Further heating (230–430°C) corresponded to a larger mass loss (about 45%). This is related to the decomposition of polymeric material linked to the support surface (L_1_/L_2_). The heterogeneity of the bio-composite surface (artificial lignin) is confirmed by the different losses in this temperature range. However, an important information provided by these results is the increased thermo-stability as effect of the covering the support with synthetic lignin. This is confirmed by the shift of the thermal effects to higher temperatures in accordance to literature data (Brebu et al., 2013; Brostow et al., 2016; Strzemiecka et al., 2016).

**Figure 3:**
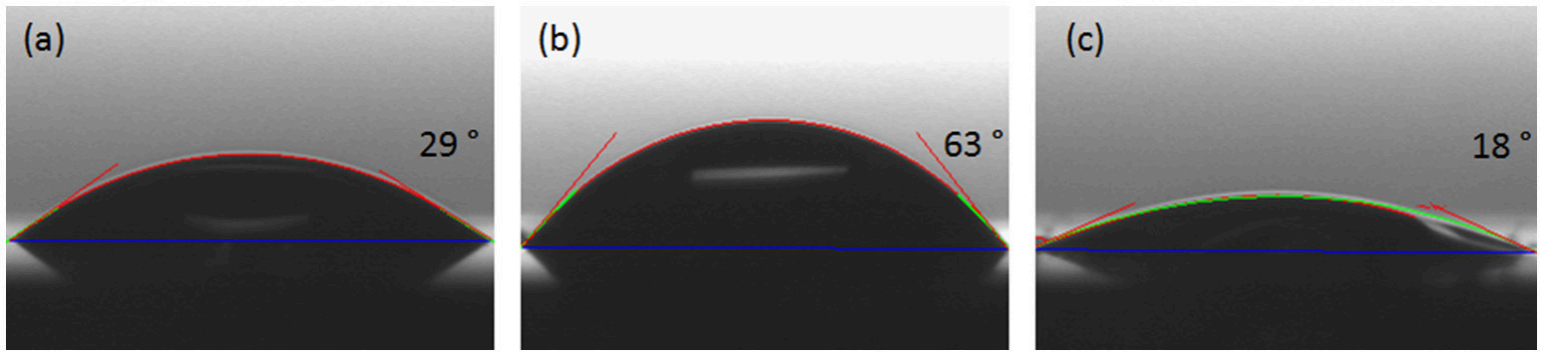
Measurements of static contact angle of **(a)** S-CafAc, **(b)** S-CafAc-L_1_, and **(c)** S-CafAc-L_2_.

The investigation of surface hydrophobicity was performed using contact angle measurements of distilled water onto CafAc-functionalized silica chips (S-CafAc) and corresponding bio-composites (S-CafAc-L_1_/L_2_) (Figure 3). The contact angle of the chip (S) was of 29°, while for the bio-composites increased to 63° for CA-based polymer or decreased to 18° for the SA-based polymer. These results indicate an enhancement of the hydrophobicity for the chip surface covered by CA monolignol reported also in the previous literature for natural lignin (Xiong et al., 2015; Salanti et al., 2016). Moreover, the artificial lignin prepared by oxi-copolymerization is more hydrophobic than some natural lignins (e.g., soda lignin from *Triticum* sp and *Saccharumofficiarum* with a 35° contact angle) (Buono et al., 2016). SA led to a hydrophilic polymer than the support as an effect of the ratio between phenolic and alkyl OH groups. For L_1_, the hydrophobicity of the aromatic ring was enforced due to the relative low density of OH confirmed by the TPD-NH_3_ measurements (Table 4). The hydrophilicity of the L_2_ surface is also according to TPD-NH_3_ results (Table 4). The hydrophobicity/hydrophilicity property of bio-composites represents an important characteristics for their further application as support/carrier of biomolecules (e.g., immobilization of lipase enzyme on hydrophobic surface turn on the enzyme in active form; Thomas et al., 2005).

**Figure 4:**
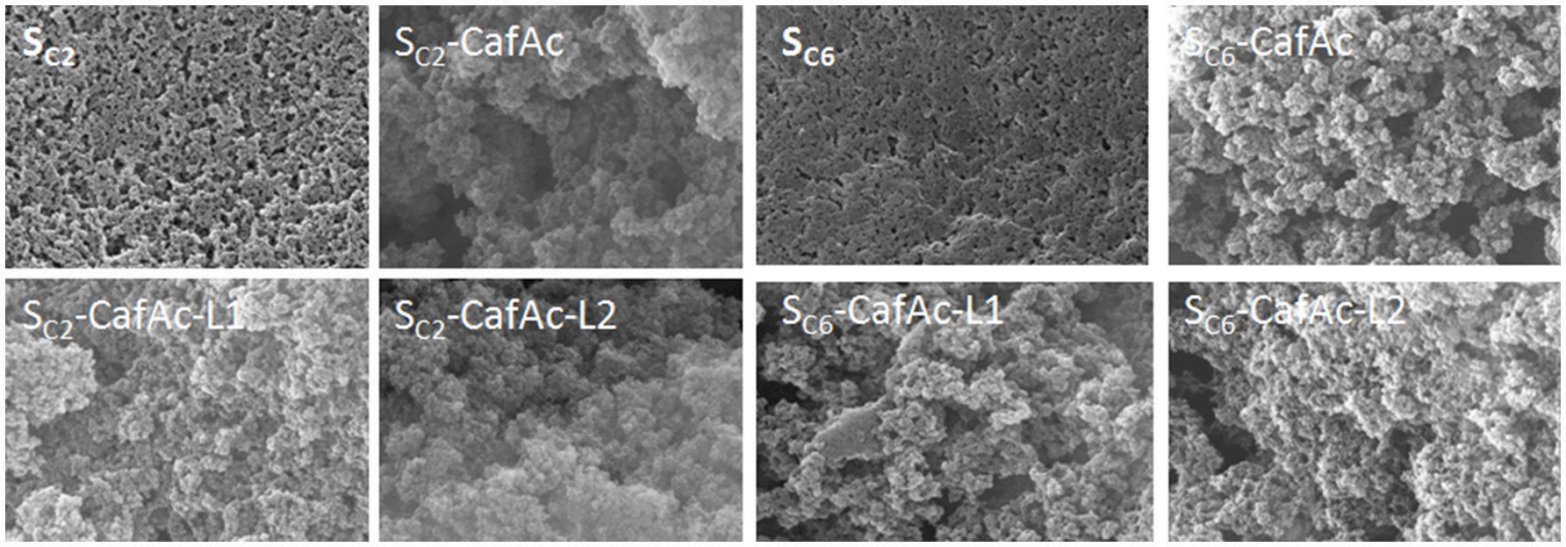
SEM images of the particles with different composition: original particles (SC2 and SC6), CafAc-functionalized particles (S_C2_/S_C6_-CafAc), and bio-composites based on CA (S_C2_/S_C6_-CafAc-L_1_), and SA (S_C2_/S_C6_-CafAc-L_2_) oxi-copolymerization.

SEM images of bio-composites and original/functionalized supports are presented in Figure 4. The parent particles (S_C2_ and S_C6_) presented a smooth surface, while the functionalized particles (S_C2_/S_C6_-CafAc) showed roughened morphology. Differences in the morphologies of the bio-composites (S_C2_/S_C6_-CafAc-L_1_/L_2_) were also induced by the monolignol oxi-copolymerization (the polymeric layer looks more dense for L_2_ than L_1_).

## Conclusions

These results confirm the success of the one-pot approach for the production of lignin bio-composites via an enzyme oxi-copolymerization process. Monolignols such as SA and CA were easily attached on a support surface based on the interaction with immobilized CafAc leading to artificial lignin covering the support surface. SA allowed an advanced polymerization (L_2_ polymer) and coverage compared to CA (L_1_ polymer). Accordingly, L_1_-based bio-composites exhibited higher hydrophobicity than L_2_, while SA provided a more acidic bio-composite surface. Therefore, the developed protocol allows the synthesis of artificial lignin-based composites with predictable surface properties.

Based on these, the reported work offers a new alternative for the valorization of lignin residues with the production of new bio-composites following a green route. The versatility of the method may offer an easy control of the properties of the prepared bio-composites and an instrument to adjust them to different applications (e.g., support/ carrier for biomolecules).

## Author contributions

All authors listed have made a substantial, direct and intellectual contribution to the work, and approved it for publication. SI and CO performed the experiments for the preparation of the bio-composites and also UV-Vis/F-C analysis; BC performed TPD-NH3 analysis; MT coordinated the research study and wrote the manuscript; IZ and AG characterized the bio-composites based on contact angle technique; AB performed FTIR analysis; ME and G-MM performed the SEM analysis and the interpretation of the corresponding results; VP revised and improved the manuscript.

### Conflict of interest statement

The authors declare that the research was conducted in the absence of any commercial or financial relationships that could be construed as a potential conflict of interest.
