# Supplementary figures and images for: One-Pot Enzymatic Production of Lignin-Composites
Source: Front Chem. 2018 Apr 20;6:124. doi: 10.3389/fchem.2018.00124 (PMC5920407; doi:10.3389/fchem.2018.00124)

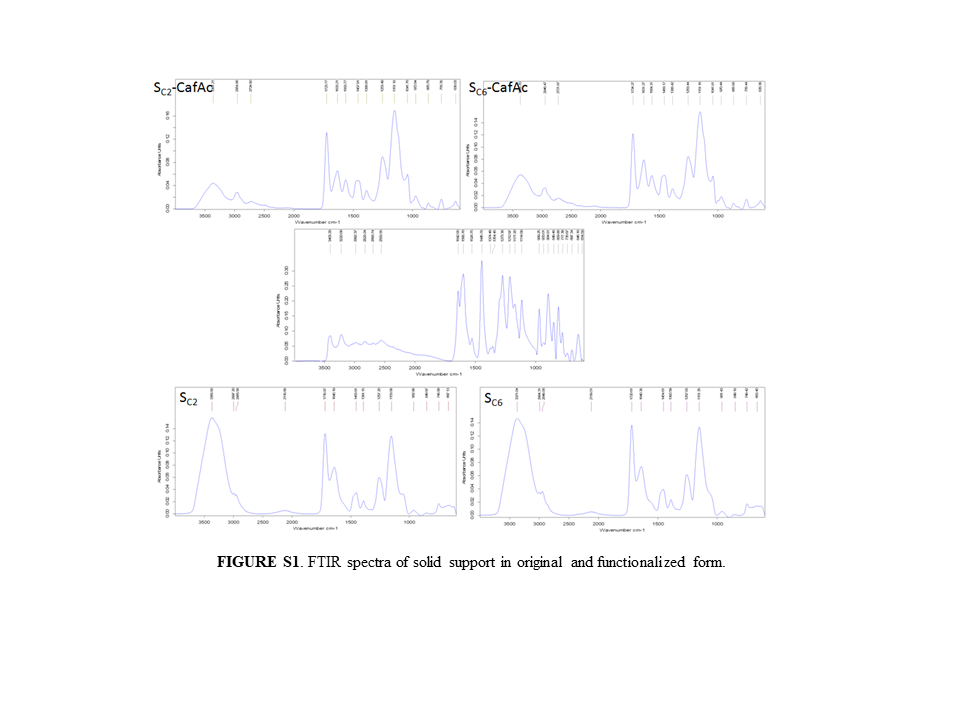

Supplement: Supplementary file 1 [file Image1.tif]

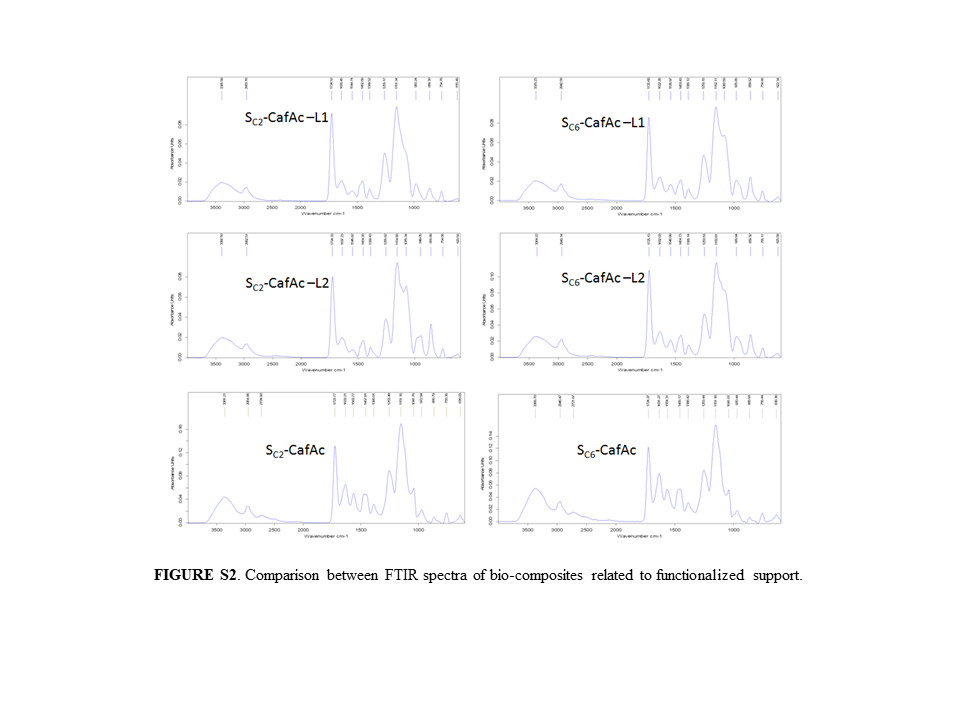

Supplement: Supplementary file 2 [file Image2.tif]

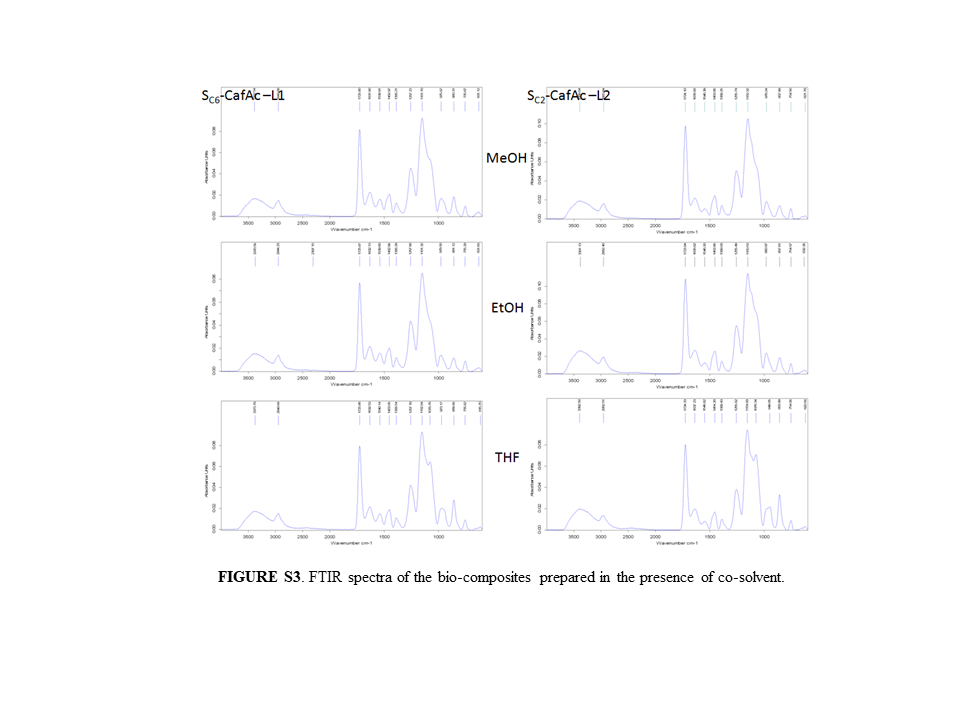

Supplement: Supplementary file 3 [file Image3.tif]
